# Effect of TGF-β_1_ Stimulation on the Smad Signal Transduction Pathway of Human Peritoneal Mesothelial Cells

**Published:** 2005-06

**Authors:** Hao Zhang, Fu-you Liu, Ying-hong Liu, You-ming Peng, Qin Liao, Ke Zhang

**Affiliations:** 1 *Department of Nephrology, Third Xiangya Hospital, Central South University, Changsha, China;*; 2 *Department of Nephrology, Second Xiangya Hospital, Central South University, Changsha, China*

**Keywords:** mesothelial cells, TGF-β_1_, Smad2/3, Smad7, extracellular matrix

## Abstract

**Objective:**

To prove whether the SMAD signal transduction pathway in human peritoneal mesothelial cells (HPMCs) influenced the process of human peritoneal fibrosis stimulated by TGF-β_1_.

**Methods:**

HPMCs were isolated from normal human omentum and the third generation cells were stimulated by 5 ng/ml TGF-β_1_. Immunohistochemistry, Western blotting, ELISA and RT-PCR were employed to investigate the protein expression of p-Smad2/3 and the protein and mRNA expressions of SMAD 7, fibronectin (FN) and collagen-I (COL1).

**Results:**

The protein expression of p-Smad2/3 in HPMCs was remarkably increased 15 min (29% p-Smad2/3-positive cells) after TGF-β_1_ stimulation, peaking from 30 min (81%) to 1 h (84%) and dropping after 2 h (37%); Meanwhile, p-Smad2/3 mainly distributed in cytoplasm at 15 min, concentrated in cell nucleus and peri-nucleus from 30 min to 1 h, and distributed in cytoplasm again at 2 h. The protein expression of SMAD 7 in cells was obviously increased 24 h after TGF-β_1_ stimulation, peaking at 48 h. The mRNA expression of SMAD7 was time-dependently increased. The expressions of extracellular FN protein, intracellular FN mRNA, as well as intracellular COL1 protein and mRNA were significantly increased and all of them displayed time dependency.

**Conclusions:**

The SMAD signal transduction pathway of HPMCs can be specifically activated by TGF-β_1_ and influence the process of human peritoneal fibrosis. The protein and mRNA expression of SMAD 7 (an inhibitor of SMAD pathway) are significantly increased as a result of feedback.

## INTRODUCTION

Ultrafiltration failure caused by peritoneal fibrosis is the major cause for patients with continuous ambulatory peritoneal dialysis (CAPD) to give up peritoneal dialysis therapy. The increase in endogenous TGF-β_1_ induced by a variety of intrinsic or extrinsic factors results in excessive accumulation of extracellular matrix around HPMCs, which contributes to the formation of peritoneal fibrosis (1, 2). Smad signaling pathway was found recently to be an important TGF-β_1_ downstream signaling pathway, which was closely related to fibrosis in many tissues such as cardiac muscle, liver, lung and kidney (3, 4). Does TGF-β_1_ dependent Smad signaling pathway exert its effect in the process of peritoneal fibrosis? However, no definite answers are available as yet. Therefore, it is necessary for us to elucidate the underlying mechanisms for the development of peritoneal fibrosis, and further to find safe and effective methods to elongate dialysis time and improve life quality of patients with peritoneal dialysis.

## MATERIALS AND METHODS

### Reagents

Goat anti-human phosphorylated Smad2/3 (P-Smad2/3) polyclonal antibody and goat anti-human Smad7 polyclonal antibody were purchased from Santa Cruz Company, USA; mouse anti-human CTGF (connective tissue growth factor) monoclonal antibody was purchased from R&D Company, USA; mouse anti-humanα-SMA monoclonal antibody, mouse anti-human COL1 (human collagen type 1) and pure TGF-β_1_ were purchased from Sigma Company, USA. ELISA kits for Plasminogen Activator Inhibitor type 1 (PAI-1) and fibronectin (FN) were purchased from Shanghai Sun Biotechnology Company. Immunohistochemistry staining kits were purchased from Beijing Zhongshan Biotechnology Co. Ltd.

### Isolation, culture and identification of HPMCs (5)

Dissection of greater omentum from healthy male adults with removal of blood vessel and lipid tissue was performed in sterile room where tissue was dissected into 1 × 1 cm^2^ pieces, washed twice with Phosphate Buffered Saline (PBS) and F12 respectively. After the tissue pieces were transferred into sterile centrifuge tube containing 20 ml 0.125% pancreatin and 0.01% EDTA, and the cultured cells were incubated on rocking bed at 250 rpm at 37°C for 15 min. The digestion was stopped by addition of 3ml 15% Fetal Calf Serum (FCS)-F12 and the samples centrifugated at 1000 rpm at 40C for 10 min. The pellet was blown once with 5 ml F12, centrifugated at 1000 rpm at 4°C for 5 min and after removal of supernatant, 5 ml of complete medium (15% FCS-F12, 0.5 μg/ml insulin, 1 μg/ml hydrocortisone, 5 μg/ml transferring, 2 μmol/ml glutamine) was added to the cells. The cell suspension was transferred into 25 cm^2^ culture flask coated with 0.1% gelatin and containing culture media? And supplemented with 100 U/ml penicillin and 100 U/ml streptomycin. The culture flasks were placed into incubator at 37°C, 5% CO_2_ for cell culturing. Cell culture media was refreshed every three days. When the cultured cells were grown into confluence, the cells were detached with 0.125% pancreatin-0.01% EDTA and plated. The third passage of the cells was used in experiments. 95% of the cells of the third passage were found to posses’ characteristics of mesothelial cells by inverted microscopy, transmission electron microscopy and scanning electron microscopy. Cytokeratin and VIII factor immunohistochemistry staining.

### Immunohistochemistry analysis of intracellular Smad2/3 phosphoralation

The confluent cells of the second passage were adjusted to the concentration of 1 × 10^5^/ml, and plated on cover glass, which was placed in 24-well culture plate. After cells reached confluence, 0.1% FCS-F12 was added, synchronized for 24 h, and after they entered the resting stage the cells were divided into 0, 15 min, 30 min, 1 h, 2 h and incubated with 5 ng/ml TGF-β1. After specified periods of time the supernatant was removed and the cover glass was taken out and fixed with pure acetone. SP method was used in p-Smad2/3 immmohistochemicalstaining (1:100). In control samples PBS was added instead of primary antibody. The translocation of p-Smad2/3 from membrane to nucleus was observed. Positive rate was calculated by counting the positive cells in 1000 cells selected randomly in each slice, and four slices were counted for each stage to get the mean value.

### Western blot assay of intracellular Smad7, CTGF, α-SMA, COL1 protein expression

The confluent cells of the second passage were adjusted to the concentration of 1 ×10^5^/ml, plated into 25 cm^2^ culture flask. After reaching confluence, the cells were cultured in 0.1% FCS-F12 and synchronized for 24 h. After reaching the resting stage, the cells were divided into 0, 15 min, 30 min, 1 h and 2 h group and incubated with 5 ng/ml TGF-β1 for specified time, for p-Smad2/3 measurements. After removal of supernatant, The cells were washed twice with PBS, and lysed with protein lysis buffer at 40C for 60 min. centrifugated at 12,000 rpm for 15 min and the resulting supernatant preserved at -70°C. 50ug total protein was electrophresed on 10% SDS-PAGE gel and transferred to nitrocellulose membrane. The membrane was blocked with blocking buffer containing 3% bovine serum albumin (BSA) and 5% skim milk powder at room temperature, incubated with anti p-Smad2/3 phosphorylated polyclonal antibody, Smad7 antibody and COL1 antibody, and the result of immune reaction was detected with ECL agent.

### ELISA assay of FN level

The confluent cells of the second passage were adjusted to the concentration of 1 × 10^5^/ml, and plated into 24-well culture plate for cell culturing as described above. After reaching confluence, 0.1% FCS-F12 was added, the cells were synchronized for 24 h and after entering resting stage divided into 0, 24 h, 48 h, 72 h groups and incubated with 5ng/ml TGF-β1 for specified periods of time. The supernatants were collected and preserved at -20°C. FN in supernatant content was determined according to the manufacturers’ instruction (Shanghai Sun Biotechnology Inc.).

### Detection of Smad7, FN and COL1 intracellular gene expression by RT-PCR

The confluent cells of the second passage were adjusted to the concentration of 1 × 10^5^/ml, divided into 25 cm^2^ culture flask for cell culture. After reaching confluence, 0.1% FCS-F12 was added and the cells were synchronized for 24 h. After entering the resting stage the cells were divided into 0, 24 h, 48 h, 72 h groups and incubated with 5 ng/ml TGF-β1 for specified periods of time. The total RNA was extracted according to the method described in instruction for Trizol reagent (GibcoBRL Inc., USA). RNA OD values were determined by ultraviolet spectrophotometer and wavelength pair of 260/280 nm. OD values ranged from 1.7 to 2.0. Three ribosome RNA strips of 28 s, 18 s and 5 s were clearly recognized on 1.2% agarose gel electrophoresis, suggesting that no pollution and degradation took place in total RNA isolation. The first cDNA was synthesized by reverse transcription according to the instruction for reverse transcription kit (Promega Inc.), which was then used as a template for PCR reaction. Primer sequences (synthesized by Shanghai Boya Biotechnology Co. Ltd.) are shown in Table 1. The reaction condition: pre-denaturation at 950 C for 5 min, denaturation at 95°C for 30 s, annealing at 56°C for 30 s, elongation at 72°C for 50 s, followed by 21 cycles for FN and COL1, and 27 cycles for smad7, elongation at 72°C for 10 min, terminated 4°C. The experiments using the cycles did not yet reach the plateau phase of PCR. PCR products were isolated by 6% polyacrylamide gel electrophoresis. Gel scanning and analysis was performed on Gene Genius image analyzer (Syngene Inc). The ratio of optical density of objective fragment to that of GAPDH fragment acting as control was used in semi-quantification comparison.

**Table 1:** Primer sequence

Primer	Sequence	Length of PCR products (bp)

Smad7-F	5’-AGC AGG CCA CAC TTC AAA CT-3’	375
-R	5’-CAC GTT GTC TCC CCA TCT G-3’	
FN-F	5’-AGC CGC CAC GTG CCA GGA TTA C-3’	439
-R	5’-CTT ATG GGG GTG GCC GTT GTG G-3’	
COL1-F	5’-AGG CTG GTG TGA TGG GAT T-3’	544
-R	5’-GGA GAG CCA TCA GCA CCT TT-3’	
GAPDH-F	5’-CAT GGG TGT GAA CCA TGA GA-3’	695
-R	5’-ACT GAG TGT GGC AGG GAC TC-3’	

### Statistical Analysis

Experimental results were expressed as mean ± standard deviation (x ± SD). One-way ANOVA or T test were used for statistical analysis using SPSS11.0 statistical software package and least significant difference method to analyze the means further.

## RESULTS

### Effect of TGF-β1 on intracellular

**Figure 1:**
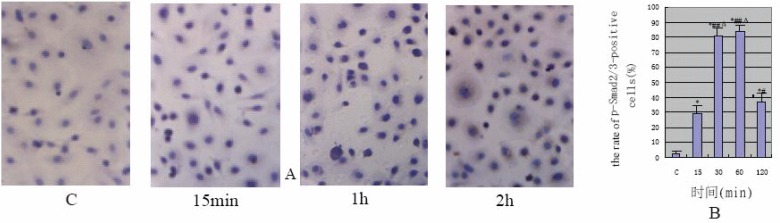
A, The protein expression of p-Smad2/3 displayed by immunohistochemistry (×400); B, TGF-β1-induced the rate of p-Smad2/3-positive cells (*P<0.01 vs C group; #*P*<0.05, ##*P*<0.01 vs 15 min group; Δ*P*<0.01 vs 120 min).

**Figure 2:**
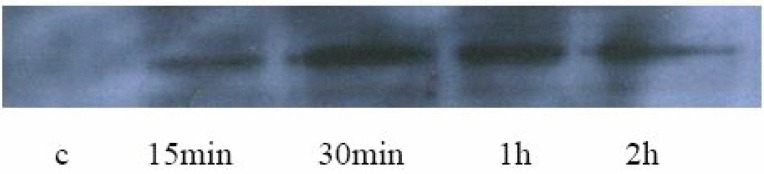
The protein expression of p-Smad2/3 detected by western blotting.

The results of immunochemical (Fig. 1) were accord with Western blotting's (Fig. 2). The HPMCs hardly expressed p-Smad2/3 protein in the control group and the cells stained lightly (the positive rate: 3% ± 1.7%). After TGF-β1 stimulation of HPMCs Smad2/3 was significantly activated at 15min (the positive rate: 29% ± 5.8%), and staining was distributed in cytoplasm; its activity peaked between 30min and 1h (the positive rate: 81% ± 5.0% and 84% ± 3.7% respectively). At that time the stain was deepened and distributed in nucleus or peri-nucleus. The phosphorylation activity was obviously reduced at 2 h (the positive rate: 37% ± 5.7%), and staining was lightened and distributed in cytoplasm again. Compared with the control, *P* value was less than 0.01.

### TGF-β1 effect on Smad7 protein and gene expression in HPMC

**Figure 3:**
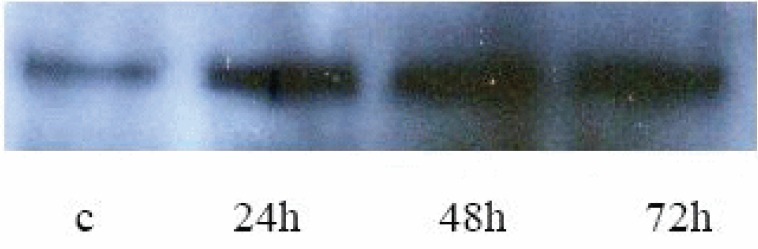
Western blot Aanalysis of Smad7.

The results of Western blot analysis (Fig. 3) the expression of Smad7 in HPMCs was expressed at a low level in control group, and increased at 24 h, peaked at 48 h after TGF- β1 stimulation of HPMC, and then decreased at 72 h.

**Figure 4:**
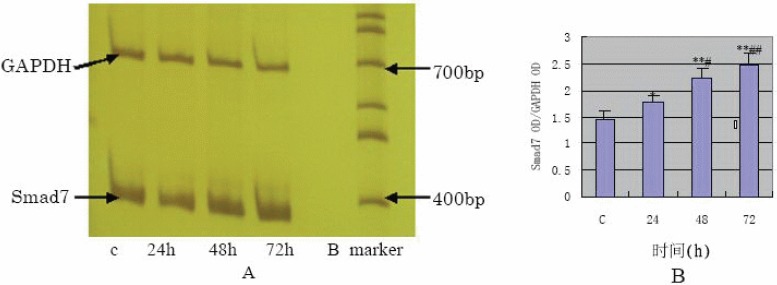
A, The mRNA expression of Smad7 detected by RT-PCR; B, The semi-quantification result of Smad7 OD/GAPDH OD (*P<0.05, **P<0.01 vs C group; #*P*<0.05, ##*P*<0.01 vs 24 h group).

The results of semi-quantification RT-PCR (Fig. 4) of TGF- β1 stimulated HPMC have shown that mRNA expression of Smad7 was increased compared with the control (*p* value <0.05 at 24 h, <0.01 at 48 h and 72 h) in a time-dependent manner.

### TGF-β1 effect on FN, COL1 protein and gene expression in HPMC

**Figure 5:**
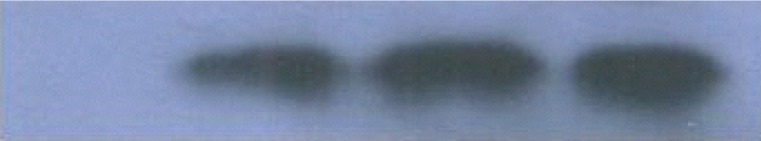
The protein expression of COL1 detected by western blotting.

**Figure 6:**
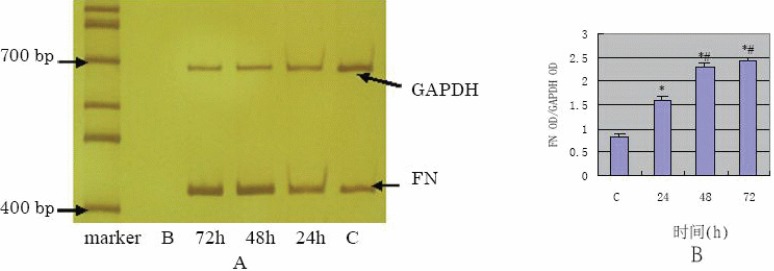
A, The mRNA expression of FN detected by RT-PCR; B, The semi-quantification result of FN OD/GAPDH OD (*P<0.01 vs C group; #*P*<0.01 vs 24 h group).

**Figure 7:**
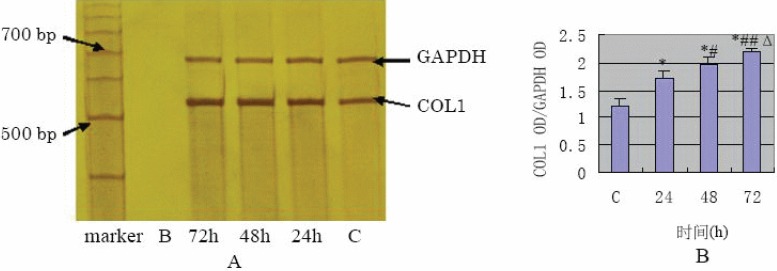
A, The mRNA expression of FN detected by RT-PCR; B, The semi-quantification result of COL1 OD/GAPDH OD (*P<0.01 vs C group; #*P*<0.05, ##*P*<0.01 vs 24h group; Δ*P*<0.05 vs 48h group).

The results of Western blot analysis (Fig. 5) have shown that COL1 showed very limited expression in the control, increased at 24 h in a time-dependent manner after TGF- β1 stimulation of the cells. ELISA assay showed FN protein expression of supernatants at 24 h, 48 h, 72h were 2.79 ± 0.44 mg/L, 3.11 ± 0.48 mg/L, 3.55 ± 0.52 mg/L respectively and the results were in a time-dependent manner compared with the control (0.67 ± 0.07 mg/L and *p* value <0.01). The results of semi-quantification RT-PCR of TGF- β1 stimulated HPMC (Fig. 6 and Fig. 7) have shown that mRNA expression of FN and COL1 was increased at 24 h (*p* value<0.01) compared with the control, also in a time-dependent manner.

## DISCUSSIONS

TGF-β1 has been well known as one of the most important trigger factor in tissue fibrosis. Does TGF-β1 have the same effect on the peritoneal fibrosis? In this study, when the third passage of HPMCs cells from its primary cultures were stimulated with TGF-β1, the subsequently obtained Western blot assay showed that the production of FN and COL1 proteins significantly increased in a time-dependent manner comparing to the control. The results of ELISA indicated that FN and COL1 protein levels in supernatants obtained from TGF-β1-stimulated cells were significantly elevated after 24h of stimulation. This effect was time-dependent and compared to the control group significantly elevated at the *p*<0.01. This data are similar to the amplification data of RT-PCR for FN and COL1 mRNA. The up-regulation of FN and COL1, the major components of extracellular matrix, demonstrates that ECM components deposit and because of excessive accumulation result in peritoneal fibrosis. Therefore, our experiments have demonstrated that TGF-β1 plays an important trigger role in peritoneal fibrosis.

There have been many researches that have attempted to block the expression or activity of TGF-β1 from different aspects, in an effort to reduce the accumulation of extracellular matrix and prevent tissue fibrosis. However, TGF-β1 which is one of the growth factors with the most complicated function that have been found so far has a wide range of biological activity on development, proliferation, differentiation, apoptosis and immune function of a variety of cells, TGF-β1 has also many side effects such as loss of cell growth control, immune disturbance, severe inflammatory and even death that will unavoidably occur if expression or activity of TGF-β1 is to be inhibited as in treatment of tissue fibrosis As a result, its application in clinical practice is limited (6). Recently, with the advances in TGF-β1 downstream signaling pathway research, the regulatory mechanism of TGF-β1 in tissue fibrosis is gradually being recognized, and inhibition or blockade in TGF-β1 downstream signaling pathway and its effective mediators have become the focus of research in prevention of tissue fibrosis.

Smad signaling pathway plays a very important role in TGF-β-induced tissue fibrosis: 1) SB-431542, a specific blocker for Smad signaling pathway, is able to suppress the TGF-β1 induced synthesis of extracellular matrix FN and COL1A1 (7); 2) Smad heterogenous oligomer binding site (SBE) exists in promoters of many genes such as FN, COL1, COL3, COL6, COL7 and COL13, TGF-β can induce extracellular matrix synthesis through Smad signaling pathway (3, 4). Moreover, SBE structure is also found in promoter of other genes including CTGF (8), TIMP-1 (3), α-SMA (9), PAI-1 (10) and Smad7 (10) which are closely related to tissue fibrosis; 3) During the course of interaction with other signaling pathway, Smad pathway is of great importance because other signaling pathways as it either interveneswith the transmission of Smad pathway or cooperate with Smad heterogenous oligomer to regulate target gene expression through its specifically activated transcriptional factor (11); 4) although little is known about whether specific blockade of Smad signaling pathway can reduce the side effects caused by direct blockade of TGF-β> Recent studies suggest that selective blockade of Smad signaling pathway and p38MAPK pathway by small protein SB-505124 can not only inhibit the synthesis of extracellular matrix but also prevent the cell death induced by direct blockade of TGF-β(12).

Does TGF-β1 specially suppress Smad signaling pathway in HPMCs? Smad2/3 is the receptor-regulated Smads and its activation by phosphorylation is the most important step in the Smad signaling pathway. Therefore, the expression of p-Smad2/3 implicates the extent to which Smad signaling pathway is activated (13). In our study, it was proven by both immumohistochemical staining and Western blot assay that the expression of p-Smad2/3 was increased at 15min after TGF-β1 stimulation, peaking at 1h and simultaneously, the increased p-Smad2/3 translocated from membrane to cytoplasm then bounded to Smad4 and in such form entered into nucleus where it exerted the effect of inducing target genes transcription. Our results confirmed that TGF-β1 could specifically activate Smad signaling pathway in HPMCs. It was also found in our experiment that the activation of Smad signaling pathway by TGF-β1 in HPMCs could persist for long time (at least 2h) and peak at a later timethen the time observed in renal tubular epithelial cell (13).

Smad7, an inhibitory Smads, negatively regulate Smad signaling pathway (3, 4). Based on the inhibitory effect of Smad7 on Smad signaling pathway, studies have been carried out to prevent the tissue fibrosis-induced by TGF-β1 using Smad7 gene transfection. For example, transfected Smad7 gene into normal rat kidney TEC line (NRK52E cells) and found that overexpression of Smad7 resulted in marked inhibition of TGF- 1 induced Smad2 activation with the prevention of collagen synthesis (13). Similarly, the data of Torada *et al* indicated that Smad7 gene transfer via adenovirus electroporation prevents unilateral ureteral obstruction (UUO)-induced renal fibrosis (14). In this study, the Western blot analysis showed that Smad7 protein remained at a low level in the absence of TGF-β1 but that it was up-regulated remarkably 24 h after the stimulation with TGF-β1, peaking at 48 h and persisted for 72 h. The result of RT-PCR showed that the level of Smad7 mRNA was increased in a time-dependent manner with p value less than 0.05 at 24 h and less than 0.01 at both 48 and 72 h. These data demonstrate that a definite level of Smad7 protein is produced by normal HPMCs and that it act mainly by keeping the Smad signaling pathway from activation. Once TGF-β1 signaling is initiated, the expression of Smad7 gene is rapidly up regulated in a feedback manner. However, the feedback is so weak that the up-regulation of Smad7 cannot offset the TGF-β1-incuded synthesis of FN and COL1. In our studies, the difference between Smad7 protein production level and mRNA expression level may be associated with the degradation of Smad7 protein by obiquitination of activated molecules in Smad signaling pathway (15).

## References

[1] LIU Yinghong, LIU Fuyou, DUAN Shaobing (2003). Influence of huangqi on TGF2beta 1 inducing extracellular matrix secretion of human peritoneal mesothelial cell. BULL. HUNAN MED. UNIV.

[2] Ha H, Lee HB (2000). Effect of high glucose on peritoneal mesothelial cell biology [J]. Perit. Dial. Int.

[3] Verrecchia F, Mauviel A (2002). Transforming growth factor-beta signaling through the Smad pathway: role in extracellular matrix gene expression and regulation [J]. J. Invest Dermatol.

[4] Chen W, Fu X, Sheng Z (2002). Review of current progress in the structure and function of Smad Proteins [J]. Chin. Med. J. (Engl).

[5] LIU Fuyou, DUAN Shaobin, LONG Zhigao (2001). Culture and characterization of human peritoneal mesothelial cells. BULL. HUNAN MED. UNIV.

[6] Huang Haichang, Li Jingzi, Wang Haiyan (2002). Transformation of renal fibroblast to myofibroblast introduced by CTGF [J]. Science Bulletin.

[7] Laping NJ, Grygielko E, Mathur A (2002). Inhibition of transforming growth factor (TGF)-beta1-induced extracellular matrix with a novel inhibitor of the TGF-beta type I receptor kinase activity: SB-431542 [J]. Mol. Pharmacol.

[8] Chen Y, Blom IE, Sa S (2002). CTGF expression in mesangial cells: involvement of SMADs, MAP kinase, and PKC [J]. Kidney Int.

[9] Hu B, Wu Z, Phan SH (2003). Smad3 mediates transforming growth factor-beta-induced alpha-smooth muscle actin expression [J]. Am. J. Respir Cell. Mol. Biol.

[10] Hua X, Miller ZA (2000). Benchabane H, Synergism between transcriptions factors TFE3 and Smad3 in transforming growth factor-beta-induced transcription of the Smad7 gene [J]. J. Biol. Chem.

[11] Lo RS, Wotton D, Massague J (2001). Epidermal growth factor signaling via Ras controls the Smad transcriptional co-repressor TGIP. EMBO J.

[12] DaCosta Byfield S, Major C (2004). SB-505124 Is a Selective Inhibitor of Transforming Growth Factor- {beta} Type I Receptors ALK4, ALK5, and ALK7 [J]. Mol. Pharmacol.

[13] Li JH, Zhu HJ, Huang XR (2002). Smad7 inhibits fibrotic effect of TGF-beta on renal tubular epithelial cells by blocking smad2 activation. J. Am. Soc. Nephrol.

[14] Torada Y, Hanada S, Nakao A (2002). Gene transfer of Smad7 using electroporation of adenovirus prevents renal fibrosis in post-obstructed kidney. Kidney Int.

[15] Lin Haiyan, Wang Hongmei, Zhu Cheng (2003). The smad signal transmitted by TGF-. Science in China (series C).

